# Serum lipid levels are the risk factors of gallbladder stones: a population-based study in China

**DOI:** 10.1186/s12944-019-1184-3

**Published:** 2020-03-19

**Authors:** Jiwen Wang, Sheng Shen, Bo Wang, Xiaojian Ni, Han Liu, Xiaoling Ni, Rong Yu, Tao Suo, Houbao Liu

**Affiliations:** 1 Department of General Surgery, Zhongshan Hospital, General Surgery Institute, Fudan University, No. 180 Fenglin Rd, Shanghai, 200032 China; 2 Meinian Onehealth Healthcare Holding Co., Ltd, Shanghai, 200030 China; 3 Biliary Tract Disease Institute, Fudan University, Shanghai, 200032 China

**Keywords:** Gallstones, Serum lipid levels, Prevalence’s risk factor, China

## Abstract

**Background:**

Gallstones are the cause of a majority of biliary tract discomfort. Although many community-based studies have addressed the risk factors for gallstone disease (GSD), little is known about GSD prevalence and risk factors in Chinese populations.

**Methods:**

From January 2014 to January 2015, participants (*N* = 2,068,523) were recruited by Meinian Onehealth Healthcare Co., Ltd. They received a physical examination, and GSD was determined by ultrasound.

**Results:**

The prevalence of GSD was 8.1%. Risks of GSD were similar between males and females in all age groups. Risk factors for gallstones include body mass index, waist circumference, waist-to-hip ratio, and physical activity, as well as biological factors such as age, sex, and elevated blood lipid levels. Serum lipid levels of GSD were statistically different from controls in total cholesterol (TC), triglycerides (TG), high-density lipoprotein cholesterol (H-DL), low-density lipoprotein cholesterol (LDL), and apolipoprotein B (APOB). Furthermore, TC > 5.00 mmol/L, TG > 1.39 mmol/L, HDL < 1.19 mmol/L, LDL > 3.04 mmol/L, and APOB > 0.97 mmol/L were risk factors for gallstones.

**Conclusions:**

Serum lipid levels are associated with GSD. TC, TG, LDL, and APOB are risk factors, while HDL is a protective factor.

## Background

Gallstones are the common cause of biliary tract discomfort. Cholecystitis, pancreatitis, and cholangitis are the most frequent complications of GSD; thus, GSD is one of the major public health issues worldwide [1]. Most patients with GSD are asymptomatic, and approximately one-fifth of patients display symptoms after one decade of follow-up [2]. Ultrasound is the preferred check for gallstones [3]. In urban residents older than 20 years, gallstone disease rates were 3.8% in South China, and 6.1% in North China [4]. However, the data were from one local company and not included people of lower economic status.

The incidence of gallstones varies in different regions [5]. In China, the incidence of gallstones continues to rise, and they have become an important health problem [6].

Previous studies have shown that gallstone risk factors include age, gender, family history, pregnancy, diabetes, and obesity.

This cross-sectional study was designed to assess the incidence and risk factors of GSD in China.

## Methods

### Study participants

Participants (*N* = 2,068,523) were recruited from January 2014 to January 2015 by Meinian Onehealth Healthcare (Group) Co., Ltd., and received a health checkup. The sample population consisted of a series of consecutive asymptomatic subjects who had regular physical examinations, rather than those with symptomatic disease. Participants who had complete information and were less than or equal to 100 years of age included 11,33,945 men and 93,4578 women in this studied.

The main medical variables were gender, age, date of birth, tobacco and alcohol consumption, family history of cardiovascular disease, previous medical conditions, and previous surgery. Medical examinations included a study of abdominal ultrasound in all subjects as part of an assessment. The cases were defined by ultrasound diagnosis as subjects with gallstones (see below), and the control group was defined as participants with no such evidence (sensitivity and specificity > 95%) [7]. Blood samples were collected from the study participants using venipuncture in a laboratory test after a night of fasting. We used the Aeroset analysis system (Abbott Laboratories, Lake Bluff, IL, USA) to measure fasting plasma glucose (FPG), TG, TC, H-DL, and L-DL.

### Physical examination

The participants’ weights were measured while they wore light clothes and took off their shoes with an accuracy of 0.10 kg. The accuracy of height was 0.5 cm. We then calculated the body mass index (BMI) by body weight (kg) divided by height (m) squared (kg/m^2^). We measured the waist circumference (accuracy of 0.1 cm) at the midpoint between the lower border of the rib cage and the iliac crest. Three blood pressure readings were measured at intervals of 1 min and averaged as a final result.

### Diagnostic criteria

The diagnosis of gallstones was based on 3.5 MHz abdominal ultrasound (US). Ultrasound was carried out by experienced ultrasound technicians. Gallstones were defined by the presence of strong intraluminal echoes that were gravity-dependent or that attenuated ultrasound transmission (acoustic shadowing) [8].

### Statistical analysis

All statistical analyses were performed using SPSS version 19.0 (SPSS Inc., Chicago, IL, USA). The results are expressed as mean ± SD. Binary variables were expressed in terms of N and percentages. Differences between the continuous variable cases and control groups were performed using Student’s t-test. The difference between the cases and controls for all categories was compared using the χ^2^ test.

## Results

A total of 2,068,523 subjects were enrolled in this study. Their ages varied from 18 to 80 years (mean 39.3 years). Of these, 1,069,109 subjects were from North China and 879,952 were from South China. Gallstones were found in 168,092 (8.1%) subjects. Overall, 102,492 were males and 65,600 were females. The prevalence of gallstone disease increased with increasing BMI. Among 168,092 subjects with GSD, 3382 were underweight (BMI < 18.5), 68,131 were normal weight (18.5 ≤ BMI < 24), and 91,413 were overweight (BMI ≥ 24), with incidence ratios of 3.5, 7.0, and 9.8%, respectively (Table 1). The prevalence of GSD in relation to the age group, waist circumference, and WHR (waist hip rate) are also shown in Table 1.

**Table 1:** Relation of gallstone disease with demographic variables

	N	NON-GSD		GSD	
		N	%	N	%
Total	2,068,523	1,900,432	91.9	168,092	8.1
Gender					
Male	1,133,945	1,031,453	91.0	102,492	9.0
Female	934,578	868,978	93.0	65,600	7.0
Age					
18–25	267,795	260,505	97.3	7290	2.7
26–35	750,899	708,073	94.3	42,826	5.7
36–45	435,034	393,377	90.4	41,657	9.6
46–55	321,267	284,018	88.4	37,249	11.6
56–65	209,784	182,991	87.2	26,793	12.8
66–75	61,930	53,180	85.9	8750	14.1
76-	21,815	18,288	83.8	3527	16.2
Area					
North	1,069,109	993,959	93.0	75,150	7.0
South	879,952	796,999	90.6	82,953	9.4
BMI(kg/m^2^)					
< 18.5	96,669	93,287	96.5	3382	3.5
18.5–23.9	976,542	908,411	93.0	68,131	7.0
24-	935,028	843,615	90.2	91,413	9.8
Waistlin(cm)					
< 75.0	2678	2552	95.3	126	4.7
75.0–83.9	2992	2729	91.2	263	8.7
84.0–91.9	2989	2654	88.8	335	11.2
92.0-	2893	2570	88.8	323	11.2
WHR					
< 0.80	869	810	93.2	59	6.8
0.80–0.85	1002	894	89.2	108	10.8
0.86–0.89	918	796	86.7	122	13.3
0.90-	1179	977	82.9	202	17.1

Note: The sum of each variable is not the same due to missing values

The mean levels of serum lipids in those with and without GSD are presented in Table 2. Compared to the controls, the GSD cases had significantly higher mean levels of TC, TG, LDL, APOB, and HDL.

**Table 2:** Relationship between serum lipid levels and GSD

Serum lipid levels^a^	N	NON-GSD		GSD		*P*
		MEAN	SD	MEAN	SD	
TC *	1,946,981	4.84	0.98	4.98	1.00	< 0.05
TG *	1,942,917	1.53	1.30	1.67	1.33	< 0.05
HDL*	1,107,261	1.31	0.31	1.27	0.37	< 0.05
LDL*	1,104,771	2.89	0.81	3.01	0.83	< 0.05
Apo A1	92,109	1.40	0.35	1.39	0.24	> 0.05
Apo B*	90,273	0.93	0.26	0.95	0.23	< 0.05

^a^ Units of TC, TG, HDL, and LDL are mmol/L,Units of Apo A1、Apo B are g/L;

*: *P* < 0.05

The risk factors for GSD in relation to the subjects are shown in Table 3. Compared to men, fewer women suffered from gallstones (OR: 0.76, 95% CI: 0.75–0.77). The risk of gallstones increases markedly with age. Participants living in the South China region had higher risks than those in North China (OR: 1.38, 95% CI: 1.36–1.39). Being underweight was negatively correlated with the risk of gallstones (OR: 0.48, 95% CI: 0.47–0.50). The risk for the overweight and obesity population was 1.45 times that of the normal weight subjects. Compared to the group with WHR less than 0.80, the risk in the group with WHR 0.80–0.85 increased by 66%, and the group with WHR 0.86–0.89 and the group with WHR more than 0.90 increased by 1.10 times and 1.84 times, respectively.

**Table 3:** Univariate analysis of the association between risk factors and GSD

Parameters	N	OR	95%CI	
			Lower	Upper
Gender				
Male	1,133,945	1.00	/	/
Female	934,578	0.76	0.75	0.77
Age				
18–25	267,795	1.00	/	/
26–35	750,899	2.16	2.11	2.22
36–45	435,034	3.78	3.70	3.88
46–55	321,267	4.69	4.57	4.81
56–65	209,784	5.23	5.10	5.37
66–75	61,930	5.88	5.69	6.07
76-	21,815	6.89	6.60	7.19
Area				
North	1,069,109	1.00	/	/
South	879,952	1.38	1.36	1.39
BMI(kg/m^2^)				
< 18.5	96,669	0.48	0.47	0.50
18.5–23.9	976,542	1.00	/	/
24-	935,028	1.45	1.43	1.46
WHR				
< 0.80	869	1.00	/	/
0.80–0.85	1002	1.66	1.19	2.31
0.86–0.89	918	2.10	1.52	2.92
0.90-	1179	2.84	2.09	3.85
TC(mmol/L)				
< 5.18	1,285,494	1.00	/	/
5.18–6.21	494,533	1.25	1.24	1.26
6.22-	166,954	1.37	1.34	1.39
TG(mmol/L)				
< 1.70	1,371,651	1.00	/	/
1.70–2.25	258,774	1.32	1.30	1.34
2.26-	312,491	1.30	1.29	1.32
HDL(mmol/L)				
< 1.04	207,201	1.00	/	/
1.04–1.54	678,238	0.85	0.84	0.86
1.55-	221,822	0.70	0.68	0.71
LDL(mmol/L)				
< 3.37	818,909	1.00	/	/
3.37–4.13	208,882	1.29	1.27	1.31
4.14-	76,980	1.38	1.35	1.41
Apo A1(g/L)				
< 1.20	16,143	1.00	/	/
1.20–1.60	60,634	1.01	0.95	1.07
1.61-	15,296	0.95	0.88	1.03
Apo B(g/L)				
< 0.80	24,884	1.00	/	/
0.80–1.10	47,916	1.24	1.18	1.31
1.11-	17,441	1.31	1.23	1.40

Note: The sum of each variable is not the same due to missing values

The risk of GSD in subjects with TC levels of 5.18–6.21 mmol/L and more than 6.22 mmol/L were 1.25 times and 1.37 times higher than those with TC less than 5.18 mmol/L, respectively. Compared to the subjects with TG levels less than 1.70 mmol/L, the risk of those with TG levels of 1.70–2.25 mmol/L and more than 2.26 mmol/L increased by about 30%. Compared to those with HDL levels less than 1.04 mmol/L, the risk those with HDL levels of 1.04–1.54 mmol/L and more than 1.55 mmol/L decreased by 15 and 30%, respectively. The risk of subjects with LDL levels of 3.37–4.13 mmol/L and more than 4.14 mmol/L were 1.29 and 1.38 times higher, respectively, than those with LDL levels of less than 3.37 mmol/L. APOA1 did not affect the incidence of GSD. The risk of those with APOB levels of 0.80–1.10 g/L and more than 1.11 g/L increased by 24 and 31%, respectively.

The results of multivariate analysis of the relationship between blood lipid levels and gallstones are shown in Table 4. HDL was a protective factor for gallstones. Compared to the subjects with HDL levels of 1.19–1.32 mmol/L, the risk of subjects with HDL levels of 1.33–1.54 mmol/L and more than 1.55 mmol/L decreased by 6 and 13%, respectively. The risk of subjects with HDL levels of 1.04–1.18 mmol/L and less than 1.04 mmol/L increased by 6 and 9%, respectively. LDL was also a risk factor for GSD. Compared to subjects with LDL levels of 2.65–3.04 mmol/L, the risks of those with LDL levels of 3.05–3.53 mmol/L and more than 3.54 mmol/L increased by 4 and 10%, respectively. The risks of subjects with LDL levels of 2.21–2.64 mmol/L and less than 2.21 mmol/L increased by 2 and 6%, respectively. Multivariate analysis showed no significant association between TC, TG, APO A1, APO B, and the incidence of gallstones.

**Table 4:** Multivariate analysis of the association between risk factors and GSD

Serum lipid levels	NON-GSD	GSD			
	N	N	OR^a^(95%CI)	OR^b^(95%CI)	OR^c^(95%CI)
TC(mmol/L)					
< 5.18	1,186,736	98,758	1.00	1.00	1.00
5.18–6.21	447,942	46,591	1.06(1.05,1.07)	1.06(1.04,1.07)	1.05(1.03,1.06)
6.22-	149,917	17,037	1.04(1.03,1.06)	1.05(1.04,1.07)	1.04(1.02,1.06)
TG(mmol/L)					
< 1.70	1,265,893	105,758	1.00	1.00	1.00
1.70–2.25	233,046	25,728	1.16(1.14,1.17)	1.11(1.09,1.13)	1.06(1.04,1.08)
2.26-	281,819	30,672	1.14(1.12,1.15)	1.07(1.06,1.09)	1.00(0.99,1.02)
HDL (mmol/L)					
< 1.04	185,396	21,805	1.00	1.00	1.00
1.04–1.54	616,617	61,621	0.85(0.84,0.87)	0.88(0.87,0.90)	0.92(0.91,0.94)
1.55-	205,022	16,800	0.68(0.66,0.69)	0.74(0.72,0.75)	0.81(0.79,0.83)
LDL(mmol/L)					
< 3.37	749,823	69,086	1.00	1.00	1.00
3.37–4.13	186,656	22,226	1.14(1.12,1.16)	1.13(1.11,1.15)	1.10(1.08,1.12)
4.14-	68,306	8674	1.15(1.12,1.17)	1.14(1.12,1.17)	1.12(1.09,1.15)
Apo A1(g/L)					
< 1.20	14,528	1615	1.00	1.00	1.00
1.20–1.60	54,515	6119	0.97(0.92,1.03)	1.01(0.95,1.07)	1.05(0.98,1.11)
1.61-	13,832	1464	0.86(0.80,0.93)	0.93(0.86,1.01)	0.96(0.88,1.04)
Apo B(g/L)					
< 0.80	22,751	2133	1.00	1.00	1.00
0.80–1.10	42,926	4990	1.11(1.05,1.17)	1.09(1.03,1.15)	1.07(1.01,1.14)
1.11-	15,531	1910	1.08(1.01,1.15)	1.06(0.99,1.13)	1.08(1.00,1.15)

^a^ Age-adjusted

^b^ Age, gender-adjusted

^c^ Age, gender, area, and BMI-adjusted

## Discussion

In China, there were few studies on the prevalence of gallstones, with inadequate sample sizes and insignificant results. We designed this cross-sectional study with a large sample size from the Chinese population. We found that the prevalence of gallstones was 8.1% (9.0% in men and 7.0% in females), slightly lower than previous figures, but slightly higher than hospital-based studies reported by Sichuan University West China Hospital, which were 10.7% (men accounted for 9.9%, and women accounted for 11.6%) [9]. A population-based screening study conducted in China in 2012 showed that the overall prevalence increased by 3.8% in South China and 6.1% in North China [4].

According to current epidemiological studies, the incidence of GSD in women in Western society is higher than that of men, and estrogen is considered an important contributor [10]. However, in our study, the incidence of GSD in males was higher than that in females. In fact, gender as a risk factor for cholelithiasis remains controversial. Although most Western studies have shown that women are more likely to develop cholelithiasis than men [11], Asian studies have not yet determined the association between the incidence of gallstones and gender [12, 13]. Liu et al. [14] found that the incidence of male cholelithiasis was higher than that of women under 50 years old, but the incidence in women over 50 years of age was higher than that of men. Hung et al. [15] noted that menopause is a risk factor for women with cholelithiasis. In addition, women’s cholelithiasis is not as significant in Asia, where pigmented stones are more common [16]. Thus, due to race or eating habits, the incidence of GSD in Asian men is higher, and most women with GSD are premenopausal and under 50 years of age.

In this population-based case-control study, high serum LDL levels and low levels of HDL were significantly associated with an increased risk of biliary calculus. This finding supports the role of serum lipids in gallstones and biliary carcinogenesis, which is consistent with previous cross-sectional and prospective studies of serum lipid and gallstones showing that high TG and low cholesterol levels are associated with gallstone risk [17–21].

Paradoxical to the generally accepted association between hyperlipidemia and gallstones, we observed that lower levels of TC, LDL, and APOB, characteristic of hypolipidemia, were also associated with biliary stones independent of the other lipids and risk factors we examined. TC, L-DL, and high levels of diabetes B were not associated with gallstones. Previous studies on the relationship between cholesterol and LDL and gallstones were contradictory, and several cross-sectional studies were reported as reverse [22, 23], positive [24–26], and ineffective [27, 28], Several prospective studies of gallstones were not associated with TC and/or low-density lipoprotein [29, 30]. The reasons for these inconsistencies are unclear but may be due to differences in research design, research population, lipid measurement methods, or mixed control deficiencies.

Our findings show to a certain extent the incidence and basic characteristics of domestic cholelithiasis as well as some common risk factors. There were some limitations to this study. The subjects of this study had physical examinations, which may differ from the general population, as people with more obvious symptoms of cholelithiasis may go directly to the hospital.

## Conclusions

It is founded in our research that serum lipid levels are associated with GSD. TC, TG, LDL, and APOB are risk factors, while HDL is a protective factor. Further studies should be conducted to understand how these factors affect cholelithiasis.

## Data Availability

The datasets used and analyzed during the current study are available from the corresponding author on reasonable request.

## Abbreviations

APO A1: Apolipoprotein A1
APO B: Apolipoprotein B
BMI: Body mass index
GSD: Gallstone disease
H-DL: High-density lipoprotein cholesterol
LDL: Low-density lipoprotein cholesterol
TC: Total cholesterol
TG: Triglycerides
WHR: Waist hip rate
